# The Highbury Hospital Fire

**Published:** 1923-11

**Authors:** 


### HIGHBURY HOSPITAL FIRE.

THE Departmental Committee of Inquiry into Fire Precautions in Ministry of Pensions Hospitals has issued its Report on the Fire at Highbury Hospital, Birmingham. (Stationery Office, 3d. net.) The Committee are of opinion that in any normal emergency the facilities provided for the removal of patients would have been ample. The progress of the fire was, however, extraordinarily rapid.

### How the Fire was Caused.

The actual cause of the fire was made clear at the inquest on the two pensioners who lost their lives. A tin of floor polish was melted over a gas boiler in a small annexe at one end of the hut. The mixture apparently caught fire and an orderly endeavoured to remove the tin from the top of the boiler :—

> The tin, however, dropped from his hands, and it was then too late to avert a disaster. The polish, now reduced to a liquid state, streamed on to the floor, blazing fiercely. It penetrated between the floor crevices, and thus, assisted by the strong wind, a fire was raging in a few moments both above and below the floor of the hut, and in an incredibly short space of time the ward was completely gutted.

### Bedridden Patients.

The Committee have endeavoured to frame recommendations for minimizing the risks of fire in hutted hospitals. The erection of permanent brick or stone buildings is out of the question as the character of the Ministry's hospital requirements is purely temporary. With reference to the removal of bedridden patients in an outbreak of fire the Committee write :—

> While not desiring to dogmatize on the subject, we are of opinion that bedridden patients should be carried out on the mattresses on which they lie, or in the case of those who could be lifted, on stretchers, of which at least two should be kept in every ward where there are any number of bedridden patients. In any ward in which there are a few bedridden cases, they should be placed immediately adjacent to a central exit, or to the exit farthest from the main corridor.

### Precautionary Measures.

It is suggested that every hospital should possess a trained fireman in charge of fire arrangements, one such fireman to be on duty both day and night. Fire alarms should be louder and more prolonged. The number of hydrants is sometimes insufficient, and enough hose should be installed in a weather-tight box to reach any portion of the hospital which the particular hydrant covers. The hydrants should be situated at intervals of not more than 150 feet apart :—

> We have had occasion to notice that heated pipes and boilers are sometimes placed in contact with woodwork. We consider that wherever possible such pipes, etc., should be at least 1 inch from any woodwork, and that where this is impossible fire-resisting non-conducting material should be inserted between the two. Where huts are heated by slow combustion stoves, and the roofs are of non-fireproof material such as rubberoid, spark catchers should be fitted to the chimneys. The storage of goods in contact with, over or near pipes should be discontinued. When coal or coke stoves are in close proximity to the walls of buildings, such walls should be suitably protected by fire-resisting non-conducting material. As regards gas apparatus, we recommend that all rubber connections should be eliminated and gas rings permanently fixed.

